# Editorial: Oxytosis/ferroptosis: unraveling the mechanisms and its multifaceted role in neurodegenerative diseases

**DOI:** 10.3389/fnmol.2024.1547301

**Published:** 2025-01-13

**Authors:** Nawab John Dar, David Soriano Castell, Shahnawaz Ali Bhat, Pamela Maher

**Affiliations:** ^1^Cellular Neurobiology Laboratory, The Salk Institute for Biological Studies, La Jolla, CA, United States; ^2^Department of Zoology, Aligarh Muslim University, Aligarh, India

**Keywords:** oxytosis, oxytosis/ferroptosis, neurodegenerative disease, Alzheimer's disease, Parkinson's disease

Oxytosis is a form of regulated non-apoptotic cell death characterized by glutathione depletion, reactive oxygen species (ROS) production, lipoxygenase activation, and calcium influx. It was first described in neuronal cells about 30 years ago and is distinct from apoptosis. The molecular mechanisms underlying this process are remarkably similar, if not identical, to the cell death process named ferroptosis some years later, when the involvement of iron-dependent lipid peroxidation was first described, and both pathways are currently thought to be one and the same process. Oxytosis/ferroptosis has emerged as a potential key factor in the progression of neurodegenerative diseases, linking various mechanisms such as oxidative stress, mitochondrial dysfunction, and immune dysregulation, and it likely plays an important role in these interconnected pathways, making it a promising target for therapeutic innovation. Recent studies have illuminated the roles of ferroptosis-related genes and molecular mechanisms in the development of Alzheimer's disease (AD) and Parkinson's disease (PD), offering potential avenues for intervention. This editorial presents four significant studies that explore the relationship between ferroptosis, immune system infiltration, and mitochondrial health in the context of neurodegenerative diseases.

Zhao et al. investigated the link between ferroptosis and AD using a robust bioinformatics approach. By analyzing datasets from the Gene Expression Omnibus (GEO) and leveraging the FerrDb database, they identified 18 differentially expressed ferroptosis-related hub genes. A machine learning-based diagnostic model using the random forest algorithm demonstrated strong predictive accuracy, with an area under the curve (AUC) of 0.824 in the training set and 0.734 in the validation set. Additionally, Zhao et al. uncovered significant alterations in the immune microenvironment of AD patients, such as increased CD4+ T resting memory cells, M2 macrophages, and neutrophils. Notable correlations were identified between ferroptosis-related hub genes and immune cells, with DDIT4 strongly linked to CD4+ T memory resting cells and AKR1C2 positively correlated with M2 macrophages. These findings not only implicate ferroptosis in AD progression but also suggest its potential as a biomarker for diagnosis. Therapeutic targets were identified by integrating microRNA (miRNA) and drug interaction analyses, with DDIT4 emerging as a promising candidate for modulating immune responses. This study highlights the intricate relationship between ferroptosis, immune dysregulation, and AD pathology, laying a foundation for future targeted therapies.

Sun et al. expanded on this topic by identifying five key ferroptosis-related genes—DDIT4, MUC1, KLHL24, CD44, and RB1—and elucidating their mechanistic roles in AD. Using advanced bioinformatics tools such as weighted gene co-expression network analysis (WGCNA) and support vector machine-recursive feature elimination (SVM-RFE), they demonstrated the involvement of these genes in autophagy and glutathione metabolism, processes central to oxidative stress regulation in neurodegeneration. Their immune profiling revealed reduced CD8+ T cells and increased populations of regulatory T cells (Tregs), macrophages, and mast cells in AD patients, further implicating some aspects of the ferroptotic pathway in immune modulation. For instance, MUC1 and KLHL24 were positively correlated with Tregs, while RB1 showed an inverse correlation with CD8+ T cells. The study also constructed a competing endogenous RNA (CeRNA) network, underscoring the regulatory complexity of ferroptosis-related genes. These findings provide a roadmap for targeting ferroptosis and its immune-related mechanisms in AD therapy.

Lyu et al. explored the convergence of ferroptosis, oxidative stress, and mitochondrial dysfunction in multiple neurological conditions, including AD, cerebral ischemia-reperfusion injury (CI/RI), and vascular dementia (VaD). They highlighted mitophagy impairment as a critical factor exacerbating oxidative damage and lipid peroxidation, thereby accelerating ferroptosis-mediated neuronal loss. In CI/RI and VaD, oxidative stress disrupts the blood-brain barrier (BBB) and cerebral vasculature, creating a conducive environment for AD pathology. Impaired perfusion further exacerbates amyloid-β accumulation, linking vascular dysfunction to neurodegeneration. Lyu et al. advocated enhancing mitophagy as a strategy to mitigate oxidative stress and ferroptosis, emphasizing the need for novel treatments targeting the intersection of these pathways. Despite advancements in acute stroke therapies such as tissue plasminogen activator (tPA) and thrombectomy, these interventions have limited efficacy in preventing long-term neurodegeneration. Similarly, FDA-approved AD drugs like memantine and donepezil address symptoms but fail to tackle mitochondrial dysfunction or ferroptosis. This underscores the necessity for therapeutic innovations targeting the root causes of neurodegeneration.

Li et al. examined the burgeoning field of ferroptosis research in PD through a bibliometric analysis. Using tools like VOSviewer and CiteSpace, they identified China as a leading contributor to global ferroptosis research and highlighted GPX4 as a central player in mitigating lipid peroxidation and oxidative stress in PD. Their findings also revealed the interplay between ferroptosis and other cell death pathways, such as autophagy and apoptosis, emphasizing the complexity of PD pathology. Immunotherapy and targeted ferroptosis modulation emerged as promising therapeutic avenues, with significant potential to improve clinical outcomes. This bibliometric overview provides valuable insights for advancing interdisciplinary research and identifying unmet needs in ferroptosis-related studies. The reviewed studies collectively emphasize ferroptosis as a pivotal mechanism in neurodegenerative diseases, providing novel insights into its interactions with immune modulation, mitochondrial dysfunction, and oxidative stress. Despite the challenges posed by the complexity of ferroptosis regulation and the heterogeneity of these diseases, these findings pave the way for the development of precision therapies. Targeting ferroptosis-related pathways has the potential to tackle unresolved in clinical challenges, potentially improving outcomes for patients with AD, PD and other neurodegenerative conditions.

In conclusion, the exploration of oxytosis/ferroptosis in neurodegenerative diseases has opened new avenues for understanding and potentially treating these complex conditions. The intricate interplay between ferroptosis, immune responses, and mitochondrial health highlights the multifaceted nature of neurodegeneration. As research progresses, the identification of ferroptosis-related biomarkers and therapeutic targets holds promise for developing precision medicine approaches that could significantly improve patient outcomes. Future studies should continue to unravel the regulatory networks and molecular mechanisms underlying ferroptosis, paving the way for innovative treatments that address the root causes of neurodegenerative diseases. By advancing our knowledge in this field, we move closer to mitigating the devastating impact of diseases like AD and PD, ultimately enhancing the quality of life for millions in an increasingly aged population.

